# Depression in middle and older adulthood: the role of immigration, nutrition, and other determinants of health in the Canadian longitudinal study on aging

**DOI:** 10.1186/s12888-019-2309-y

**Published:** 2019-11-06

**Authors:** Karen M. Davison, Yu Lung, Shen (Lamson) Lin, Hongmei Tong, Karen M. Kobayashi, Esme Fuller-Thomson

**Affiliations:** 1 Faculty of Social Science, University of Hawaii, Honolulu, Hawaii USA; 2 Faculty of Science and Horticulture (Health Science), Kwantlen Polytechnic University, Surrey, British Columbia Canada; 3 Factor-Inwentash Faculty of Social Work, University of Toronto, Toronto, Ontario Canada; 4 Faculty of Health and Community Studies, MacEwan University, Edmonton, Alberta Canada; 5 Faculty of Social Science, University of Victoria, Victoria, British Columbia Canada; 6 Institute for Life Course & Aging, University of Toronto, Toronto, Ontario Canada

**Keywords:** Older adults, middle-age, immigration, determinants of health, nutrition, CLSA, depression

## Abstract

**Background:**

Little is known about depression in middle-aged and older Canadians and how it is affected by health determinants, particularly immigrant status. This study examined depression and socio-economic, health, immigration and nutrition-related factors in older adults.

**Methods:**

Using weighted comprehensive cohort data from the baseline Canadian Longitudinal Study on Aging (*n* = 27,162) of adults aged 45–85, gender-specific binary logistic regression was conducted with the cross-sectional data using the following variables: 1) Depression (outcome) measured using the Center for Epidemiologic Studies Short Depression (CESD-10) rating scale; 2) Immigration status: native-born, recent and mid-term (< 20 years), and long-term immigrants (≥20 years); and 3) covariates: socioeconomic status, physical health (e.g., multi-morbidity), health behavior (e.g., substance use), over-nutrition (e.g., anthropometrics), under-nutrition (e.g., nutrition risk), and dietary intake.

**Results:**

The sample respondents were mainly Canadian-born (82.6%), women (50.6%), 56–65 years (58.9%), earning between C$50,000–99,999 (33.2%), and in a relationship (69.4%). When compared to Canadian-born residents, recent, mid-term (< 20 years), and longer-term (≥ 20 years) immigrant women were more likely to report depression and this relationship was robust to adjustments for 32 covariates (adjusted ORs = 1.19, 2.54, respectively, *p* < 0.001). For women, not completing secondary school (OR = 1.23, *p* < 0.05), stage 1 hypertension (OR = 1.31, *p* < 0.001), chronic pain (OR = 1.79, *p* < 0.001), low fruit/vegetable intakes (OR = 1.33, *p* < 0.05), and fruit juice (OR = 1.80, *p* < 0.001), chocolate (ORs = 1.15–1.66, p’s < 0.05), or salty snack (OR = 1.19, *p* < 0.05) consumption were associated with depression. For all participants, lower grip strength (OR = 1.25, *p* < 0.001) and high nutritional risk (OR = 2.24, *p* < 0.001) were associated with depression. For men, being in a relationship (OR = 0.62, *p* < 0.001), completing post-secondary education (OR = 0.82, *p* < 0.05), higher fat (ORs = 0.67–83, p’s < 0.05) and omega-3 egg intake (OR = 0.86, *p* < 0.05) as well as moderate intakes of fruits/vegetables and calcium/high vitamin D sources (ORs = 0.71–0.743, p’s < 0.05) predicted a lower likelihood of depression. For men, chronic conditions (ORs = 1.36–3.65, p’s < 0.001), chronic pain (OR = 1.86, *p* < 0.001), smoking (OR = 1.17, *p* < 0.001), or chocolate consumption (ORs = 1.14–1.72, p’s < 0.05) predicted a higher likelihood of depression.

**Conclusions:**

The odds of developing depression were highest among immigrant women. Depression in middle-aged and older adults is also associated with socioeconomic, physical, and nutritional factors and the relationships differ by sex. These results provide insights for mental health interventions specific to adults aged 45–85.

## Background

On a global basis, depressive disorders are estimated to impact one in every 23 people [1]. Major depressive disorder is the second leading cause of disability and accounts for one-twelfth of all years lost to disability world-wide [2]. While the etiology of the condition is multi-factorial, a particular concern is depression that occurs in adulthood as it has been associated with chronic disease incidence and complications, high utilization of health services, nursing home admission, suicide, dementia, and shortened life expectancy [3–6].

Theoretical and empirical evidence indicates that various social determinants are associated with depression. Higher prevalence or incidence of major depression tends to occur in women [7] as well as older adults, those who are not married, and those with lower incomes [8]. The association between depression and education level seems to interact with working status. Longitudinal research indicates that among those free from depression at baseline, individuals with a secondary school education or less have a much higher likelihood of developing a major depressive episode (MDE) than more educated working respondents. Among the unemployed, higher risk of major depression was found among those with a higher level of education [9].

Nutrient intake, health behaviors and health status are also related to depression. Cross-sectional and longitudinal studies that have compared intakes of grains, dairy, and lean meats or ``traditional`` diet patterns (e.g., Mediterranean, Japanese, Norwegian) and “unhealthy” diets have shown that increased adherence to low quality diets (e.g., more processed foods, nutrient poor) are less protective against depression diagnosis and symptoms [10–17]. Longitudinal investigations indicate that health behaviors such as smoking and physical activity have a larger impact on depression than diet [18]. Additional risk factors for depression include substance use, a family history of psychiatric disorders [19, 20], presence of pain [21] and co-morbidities [22], and, for women in particular, high visceral adiposity [23].

There has been limited exploration of these relationships among marginalized groups such as immigrants. Globally, substantial regional variation in the occurrence of depression exists [1] and levels among immigrants may be closer to the average of individuals in their country of origin than their receiving country. For example, Asian immigrants in Canada experience a much lower prevalence of depression than immigrants from Europe, the USA or Canada [24], which is consistent with the relative level of depression in the source countries. Among Canadian immigrants, cross-sectional studies report lower levels of depressive symptoms compared to native born Canadians [25–28]. A representative longitudinal study of new immigrants showed a steep increase over time in self-reported sadness and/or loneliness from 5.1% in the first 6 months in Canada to 30% after 2 years, a level which persisted even 4 years after arrival [29]. Several factors may confound the relationship between immigrant status and depression such as age [28, 30, 31], gender, marital status and education. Men, as well as those who are married, and those who have a higher education are less likely to experience depression [27, 32]. Other contributors to depression include chronic physical or mental health conditions, chronic pain [33, 34], substance use [35], and obesity [36]. Although recent immigrants are less likely to be overweight and obese when compared to native-born residents, the prevalence of obesity among longer-term immigrants converges with that of the native-born population [37]. Little is known about how obesity affects the level of depression among immigrants [38].

There are several reasons why the migration, integration and settlement process could contribute to immigrants’ vulnerability to mental ill health [39]. Immigrating individuals may experience substantial stress associated with settling in a new country due to insufficient income [40], language barriers [41, 42], discrimination, cultural adaptation [43], reduced social support networks [44, 45], and a lack of recognition of their education and work experiences in the receiving countries [46]. These challenges may result in downward social mobility and concomitantly a higher risk of mental health problems, especially for immigrant women [46–48]. The impact of immigration on mental health depends on both individual and societal factors such as language proficiency and health care access in the host country [49, 50]. Despite the stress of migration, on average, immigrants are in better health than their non-immigrant peers in the receiving country [51–56], particularly after socioeconomic status is taken into account [53, 57]. The health advantage of immigrants, also known as the healthy migrant effect, has been reported among select immigrant populations in the US [58, 59], Australia [60], and Canada [61]. Such favorable effects may be due to processes of positive selection for individuals without chronic or acute health problems [62] and reverse migration (i.e., the return of immigrants to their home countries once they become ill) which may falsely increase the reporting of good health among immigrants in their receiving countries [54, 59, 62].

Culture-specific dietary habits and health behaviors may contribute to good health in the early years for immigrants, however, their diets may change significantly over time resulting from negative acculturation [63]. A US study found that although newer immigrants were at a lower risk of carrying excess weight, the prevalence of obesity among American immigrants living more than 15 years was similar to that of the US-born population [64]. For Canadian immigrants, the acculturation process may be conducive to healthy dietary habits by reducing nutritional deficiencies in calcium, iron and vitamin D, but it may also contribute to poor health related to higher intakes of fat and sodium [38]. Studies of smoking behavior have indicated that in some immigrant subpopulations, particularly women, smoking rates increase after immigration [65, 66]. Whether the healthy migrant effect is maintained after decades in the host country is not clearly established. Some studies have indicated that immigrants’ health advantage appears to deteriorate over time [67–69] and may converge with native-born population levels [52], while other investigations reported that over time immigrants continue to maintain better health than native-born populations [70].

Promoting excellent mental health among middle-aged and older Canadians, both immigrants and non-immigrants, is an important priority among policymakers, health practitioners and researchers [38, 71, 72]. However, there is inadequate information on the relationship between depression in this population and immigration status, socioeconomic status, physical health (e.g., multi-morbidity), health behaviors (e.g., substance use, physical activity), over-nutrition (e.g., anthropometrics, disease risk), poor nutrition status (e.g., handgrip strength, nutrition screen scores, body composition, anemia screen), and dietary intake.

To help address these knowledge gaps, the current study utilized baseline data from the Canadian Longitudinal Study on Aging. The focus of the analysis was to explore the following research questions/objectives in middle-aged and older adults:

1. Is immigrant status associated with depression among Canadian women and/or Canadian men aged 45 to 85 years?
2. To what extent does adjustment for a wide range of demographic, social, economic, and health-related characteristics attenuate the association between immigrant status and depression?
3. Is there an association between dietary intake and depression after controlling for immigration status?
4. What other factors are associated with depression among Canadians aged 45 to 85 years after controlling for immigration status?

## Methods

### Study population

The sample of this study included the baseline comprehensive set of participants (*n* = 30,097) of the Canadian Longitudinal Study on Aging (CLSA). The CLSA is a prospective study that includes biological, medical, psychological, social, lifestyle, and economic measures of Canadians aged 45 to 85 years old who will be followed for 20 years [73]. Participants in the comprehensive sample were administered in-home interviews and a wide range of physical assessment data was collected from them at dedicated data collection sites [74]. Those who did not have their anthropometrics assessed were excluded (*n* = 2935), yielding a final sample size of 27,162. Further details about the study protocol can be found at: https://www.clsa-elcv.ca.

### Measures

#### Dependent variable

##### Depression

The outcome, depression, was measured using the Center for Epidemiologic Studies Short Depression (CESD-10) rating scale. The CESD-10 contains Likert scale questions that assess depressive symptoms in the past week. It includes 10 questions: three about depressed affect, five about somatic symptoms, and two about positive affect (e.g., hopefulness for the future). Each question has four possible response options that range from “all of the time” (5–7 days; score of 3) to “rarely or none of the time” (score of 0). Total scores range between 0 to 30 and the cut-off score of ≥10 [75] was used to screen for depression.

#### Independent variable

##### Immigration status

The primary relationships of investigative interest were the differences between foreign-born immigrants of Canada and native-born Canadians in relation to depression. Three categories of participants were compared: recent and mid-term immigrants (< 20 years), longer-term immigrants (≥20 years), and native-born Canadians.

#### Covariates

Demographic, socioeconomic, and health-related variables that could attenuate the relationship between depression and immigration status were also examined in the analysis.

##### Demographic, social and economic characteristics

Demographic measures included age (45–55, 56–65, 66–75, 76–85 years), sex (men, women), and relationship status (single/ married, living with a partner, or common-law/widowed, divorced or separated). Socioeconomic status measures included level of education (less than secondary school graduation, high school graduate or with some post-secondary education, post-secondary degree or diploma, non-response) and household income, defined as the best estimate of the total gross household income received by all household members in the past 12 months (<$20,000, $20,000 to <$50,000, $50,000 to <$100,000, $100,000 to <$150,000, ≥$150,000, non-response).

##### Physical health measures

Multi-morbidity measures were based on reported diagnosis by a doctor of at least one of 18 chronic conditions (diabetes, borderline diabetes or blood sugar, heart disease or congestive heart failure, peripheral vascular disease or poor circulation in limbs, dementia or Alzheimer’s disease, multiple sclerosis, epilepsy, migraine headaches, intestinal or stomach ulcers, bowel disorders such as Crohn’s disease, ulcerative colitis, or irritable bowel syndrome, macular degeneration, anxiety disorder, mood disorder, back problems (excluding fibromyalgia and arthritis), kidney disease or kidney failure, rheumatoid arthritis, osteoarthritis in the hands, hip and/or knee, and cancer). The number of conditions was summed and the total values were categorized as no health condition, one health condition, two health conditions and more than two health conditions. Chronic pain was a derived variable that was based on two questions about whether or not respondents were usually free from discomfort (yes/no) and the number of activities that their pain or discomfort prevented them from doing (none, few, some, most). Responses to these queries were coded as either “free from pain” or “a few, some or most activities prevented by pain”. Average blood pressure results were based on the average of five readings. With reference to established guidelines [76], the average blood pressure readings were categorized into five types: normal (systolic < 120 mmHg and diastolic < 80 mmHg), elevated hypertension (systolic 120–129 mmHg and diastolic < 80 mmHg), stage one hypertension (systolic 130–139 mmHg or diastolic 80–89 mmHg), stage two hypertension (systolic ≥140 mmHg or diastolic ≥90 mmHg), and currently taking anti-hypertensive medication.

##### Health behavior measures

Smoking status was a binary variable that was based on responses to a question pertaining to whether participants ever smoked at least 100 tobacco cigarettes in their lifetime (yes/no). Drinking behavior was classified as non-binge drinker, occasional binge drinking, and regular binge drinking. Binge drinking behavior was defined as men who had five or more drinks or women who had four or more drinks on the same occasion. The variable was categorized as regular (at least once a month in the past 12 months) [77], occasional (less than once a month but at least once in the past 12 months), and non-binge drinkers (never in the past year). Physical activity was based on a question that asked about frequency in engagement of light sports or recreational activities (e.g., bowling, golf with a cart, shuffleboard, badminton, fishing) in the previous 7 days (never or seldom, sometimes or often, non-response).

##### Nutrition status indicators

There were five body composition-related measures included in the analysis: 1) body mass index (BMI) categorized as obese (≥ 30 kg/m^2^), underweight (< 18.5 kg/m^2^), healthy weight (18.5 to 24.99 kg/m^2^), overweight (25 to 29.99 kg/m^2^); 2) waist-to-hip ratio (WHR) dichotomized into high risk and low risk based on the cut-off of 0.85 for women and 0.90 for men [78]; 3) waist-to-height ratio (WHtR) where values above 0.50 suggest an increased risk [79]; 4) body fat percent based on dual-energy x-ray absorptiometry (DEXA) and subdivided into five categories (< 26%, 26–31%, 31–36%, 36–41%, 41–59%); and 5) a composite measure of disease risk based on the standard aforementioned classifications of BMI and WHR (least risk, increased risk, high risk, very high risk) [80]. Five measures of poor nutrition status: 1) handgrip strength (HGS) measured by the Tracker Freedom wireless grip dynamometer. Based on cut-off values of 19.2 kgf (kilogram force) for women 45 years^+^ and 37.9 kgf for men between 45 and 64 years and 30.2 kgf for men 65 years^+^ [81], three categories were included: under-nutrition, no under-nutrition, or not assessed; 2) nutritional risk derived from responses to a standardized assessment tool, namely, Abbreviated Seniors in the Community Risk Evaluation for Eating and Nutrition II (AB SCREEN™ II).

Total scores were grouped into high risk (< 38), low risk (≥38), and not assessed; 3) sarcopenia based on skeletal muscle mass measured using DEXA and a derived skeletal muscle index that was derived using European Working Group on Sarcopenia in Older People’s screening algorithms [82]. Sex-specific quintile points were applied with the 20 percentile used as the cut-off score to classify respondents as having sarcopenia or not [83]; 4) bone mineral densities (BMDs) measured by DEXA that yielded T-score values: osteoporosis (T-score ≤ − 2.5), osteopenia (T-score − 1 to − 2.5), and normal bone density (T-score > − 1); 5) anemia screen based on measures of haemoglobin with the cut-off values of ≤119 g/L for women and ≤ 129 g/L for men [84]. A third category for those who did not consent to blood work was included.

##### Dietary intake measures

Dietary intake assessments were measured using a semi-quantitative 36-item Short Diet Questionnaire that evaluated habitual consumption frequencies of selected food groups (e.g., fruits and vegetables) and foods that are sources of select nutrients of interest (e.g., fats, calcium, vitamin D) as standard portions per day [85]. Fiber intake was based on consumption of high fiber breakfast cereals, whole wheat, bran, multigrain, and rye breads. Pulses and nuts included legumes (beans, peas, lentils), nuts, seeds and peanut butter. Fat sources were based on total consumption of the following food items: French fries or pan-fried potatoes, poutine; butter or regular margarine on bread or on cooked vegetables only; regular vinaigrettes, salad dressings, mayonnaise, homemade or commercial dips; beef, pork (ground, hamburgers, roast beef, steak, cubed); other meats (veal, lamb, game); patés, cretons, terrines; sauces and gravies (brown, white); sausages, hot dogs, ham, smoked meat, bacon; all egg dishes except omega 3 eggs (eggs, omelette, quiche); chicken, turkey. Omega 3 fatty acid intakes were based on consumption of fish and consumption of omega 3 eggs. Daily average intake of fruits and vegetables included fresh, frozen, or canned fruits, green salad, potatoes, carrots and other vegetables. Calcium-containing food sources with high vitamin D content were based on average daily intakes of calcium-fortified milk (35% more calcium), whole and skimmed milk (3.25, 2, 1% milk fat), low-fat and regular cheeses; milk-based desserts; calcium-fortified beverages and juices. Calcium-containing food sources with low vitamin D content included yogurt (low-fat and regular) and calcium-fortified foods. Other food groups that were assessed included 100% pure fruit juices (e.g. orange, grapefruit or tomato), salty snacks (e.g. regular chips, crackers), pastries (e.g. cakes, pies, doughnuts, pastries, cookies, muffins), and chocolate bars.

For variables where there were missing values, the Missing Indicator Method was applied [86]. Missing values included responses such as “not answered” or “refused.” It also included clinical variables that were “not assessed” and measures where consent was not provided (“no consent for sample”).

### Statistical analysis

All analyses were completed using SPSS Version 22 [87]. As a modification of the model-based approach, normalized trimmed inflation weights were applied to provide estimates that are representative of the national population. The standardized weights were derived by dividing the trimmed inflation weight of each unit used in the analysis by the (unweighted) average of the survey weights of all the analyzed units.

Descriptive information and inferential statistics were generated by Chi-square tests using weighted means. Adjusted odds ratios (aOR) were derived from binary logistic regression to examine associations between immigrant status and depression while adjusting for the covariates. Six stepwise models were analyzed by entering demographic and socioeconomic variables (Model 1), physical health measures (Model 2), health behavior variables (Model 3), over-nutrition and poor nutrition status indicators (Models 4 and 5), and dietary intake measures (Model 6) respectively. The final model was adjusted for all aforementioned variables (Model 7). The interaction variable of sex by immigration status was significant and therefore separate logistic regression analyses were conducted for women and men.

## Results

### Description of sample

Table 1 presents details about the proportion of men and women in the sample who were experiencing depressive symptoms. Overall, the sample mainly consisted of Canadian born residents (*n* = 22,423, 82.6%), 56–65 years (*n* = 16,008, 58.9%), earning between C$50,000–$99,999 annually (*n* = 9031, 33.2%), in a married or common-law relationship (*n* = 18,859, 69.4%), who had earned a post-secondary diploma or degree (*n* = 21,115, 77.7%). Among the demographic, social, and economic factors, significant associations were found for men and women by income, relationship status, and education level categories (p’s < 0.001). For the age, significant association was only found among men (*p* < 0.001).

**Table 1:** Description of CLSA Sample by Sex (*n* = 27,162)

Variable	Men (*N* = 13,417)			Women (*N* = 13,745)		
	Depressed Sample (Weighted %)	Total Sample (Unweighted n)	χ^2^ (df), *p*-value	Depressed Sample (Weighted %)	Total Sample (Unweighted n)	χ^2^ (df),*p*-value
Demographic, Social, and Economic Factors						
Immigration status						
Native-born Canadian	12.6%	10,841	2.74 (2), .255	17.1%	11,582	31.8 (2), <.001
Immigrant < 20 years	14.5%	301		28.9%	214	
Immigrant ≥20 years	11.8%	2275		18.8%	1949	
Age						
45–55 years	13.7%	3382	25.8 (3), <.001	17.4%	3615	7.22 (3), .065
56–65 years	12.8%	4349		17.4%	4662	
66–75 years	9.9%	3326		16.7%	3259	
76–85 years	10.6%	2360		19.9%	2209	
Income						
< $20,000	38.0%	462	446.4 (5), <.001	36.7%	838	399.6 (5), <.001
$20,000–$49,999	18.5%	2268		23.5%	3339	
$50,000–$99,999	13.0%	4604		16.7%	4427	
$100,000–$149,999	9.9%	2865		12.3%	2242	
≥ $150,000	6.6%	2610		10.1%	1865	
Not answered	18.1%	608		23.1%	1034	
Relationship status						
Single	24.8%	1056	295.4 (2), <.001	24.3%	1263	108.2 (2), <.001
Married/live with a partner/common-law	10.3%	10,510		15.4%	8349	
Widowed/divorced/separated	21.4%	1851		22.1%	4133	
Education level						
< secondary school	20.8%	642	77.4 (3), <.001	27.7%	811	65.7 (3), <.001
High school graduate &/or some post-secondary	16.7%	2087		19.2%	2466	
Post-secondary degree/diploma	11.4%	10,663		16.5%	10,452	
Not answered	17.6%	25		15.8%	16	
Physical Health						
Morbidities						
No health conditions	6.4%	2702	527.4 (3), <.001	8.1%	2163	586.0 (3), <.001
1 health condition	8.7%	3783		10.7%	3344	
2 health conditions	11.8%	3121		17.6%	3128	
3 health conditions	23.4%	3811		27.7%	5110	
Hypertension levels						
Normal	11.9%	4008	9.60 (4), .048	15.1%	5869	67.4 (4), <.001
Elevated	10.5%	1144		17.6%	1441	
Stage 1 hypertension	13.4%	2515		20.4%	1823	
Stage 2 hypertension	13.5%	1593		18.7%	1283	
Taking anti-hypertensive	12.8%	4157		21.4%	3329	
Chronic pain						
No pain	9.8%	10,651	376.1 (2), <.001	13.6%	9918	416.4 (2), <.001
Pain	24.4%	2175		29.1%	3206	
Refused	20.8%	591		25.9%	621	
Health Behaviors						
Smoking lifetime						
≥ 100 cigarettes	14.6%	7614	61.3 (1), <.001	18.8%	6870	14.4 (1), <.001
< 100 cigarettes	10.1%	5803		16.4%	6875	
Binge drinking						
No binge drinking	13.1%	8076	15.5 (2), <.001	18.5%	9476	15.0 (2), .001
Regular binge drinking	13.1%	2562		16.6%	1744	
Occasional binge drinking	10.5%	2779		15.5%	2525	
Physical activity						
Never or seldom	12.3%	11,588	28.8 (2), <.001	17.6%	11,676	33.9 (2), <.001
Sometimes or often	11.4%	1274		14.4%	1501	
No answer or refused	20.3%	555		25.9%	568	
Nutrition Status Indicators						
BMI						
Underweight: < 18.5	33.3%	48	47.4 (3), <.001	24.1%	144	75.7 (3), <.001
Healthy: ≥ 18.5 - < 25	11.3%	3229		14.8%	4848	
Overweight: ≥ 25- < 30	11.5%	6176		17.2%	4804	
Obese: ≥ 30	14.9%	3964		21.8%	3949	
Waist-to-hip ratio						
Low risk	10.7%	1313	4.48 (1), .034	16.1%	7951	32.6 (1), <.001
High risk	12.7%	12,104		19.9%	5794	
Waist-to-height ratio						
Below cut-off	11.2%	8446	34.9 (1), <.001	15.6%	9685	91.7 (1), <.001
Above cut-off	14.7%	4971		22.6%	4060	
Body fat percent						
0–26%	10.5%	4671	73.1 (4), <.001	15.4%	313	82.0 (4), <.001
26–31%	12.3%	4482		13.7%	944	
31–36%	14.2%	3032		14.1%	2443	
36–41%	19.6%	961		16.7%	4271	
41–59%	18.8%	271		21.1%	5774	
Disease risk						
Least risk	11.6%	3235	44.5 (3), <.001	14.8%	4729	76.5 (3), <.001
Increased	11.0%	4438		17.1%	2690	
High	12.4%	2139		17.8%	2541	
Very high	15.7%	3605		22.1%	3785	
Grip strength						
No under-nutrition	11.1%	10,111	96.3 (2), <.001	16.2%	10,713	81.8 (2), <.001
Under-nutrition	17.1%	2654		24.1%	1843	
Not assessed	20.2%	652		23.4%	1189	
Nutritional risk						
Low risk	7.7%	8452	524.5 (2), <.001	11.2%	8312	649.8 (2), <.001
High risk	21.7%	4269		28.7%	4751	
Not assessed	18.6%	696		25.3%	682	
Skeletal Muscle Index (SMI)						
No sarcopenia	18.0%	2135	57.4 (1), <.001	17.6%	13,739	0.14 (1), .706
Sarcopenia	11.7%	11,282		12.5%	6	
T-scores						
Normal	12.4%	12,421	1.58 (2), .454	17.2%	8589	3.66 (2), .161
Osteopenia	12.9%	63		19.3%	1154	
Osteoporosis	14.1%	933		18.2%	4002	
Screen for anemia						
Negative	12.0%	10,448	15.6 (2), <.001	17.4%	11,316	2.30 (2), .317
Positive	16.5%	1049		23.0%	96	
No consent for sample	13.5%	1920		18.2%	2333	
Dietary Intakes						
Average daily intakes of fiber sources						
0 to < 1	13.2%	4151	6.48 (3), .091	19.2%	4625	21.8 (3), <.001
≥ 1 to < 2	12.2%	6334		16.0%	6507	
≥ 2 to < 3	11.5%	2305		18.2%	2134	
≥ 3	14.5%	627		19.9%	479	
Average daily intakes of pulses and nuts						
0 to < 0.5	14.6%	4293	27.9 (3), <.001	19.7%	4468	20.56 (3), <.001
≥ 0.5 to < 1	12.1%	3262		16.6%	3004	
≥ 1 to < 2	11.0%	4986		16.8%	5354	
≥ 2	12.6%	876		15.5%	919	
Average daily intakes of fat sources						
0 to < 2.5	14.9%	1649	18.1 (3), <.001	19.0%	1715	4.87 (3), .182
≥ 2.5 to < 4	12.3%	5127		17.3%	5128	
≥ 4 to < 5	10.9%	3232		16.7%	3398	
≥ 5	13.2%	3409		18.1%	3504	
Intakes of fish						
No consumption	21.0%	1121	82.9 (1), <.001	22.1%	1150	17.3 (1), <.001
Consumes fish	11.7%	12,296		17.2%	12,595	
Intakes of omega-3 eggs						
No consumption	12.8%	10,099	3.18 (1), .075	18.2%	9959	9.61 (1), .002
Consumes omega-3 eggs	11.7%	3318		16.0%	3786	
Average daily intakes of fruits and vegetables						
0 to < 2	18.3%	2631	119.0 (4), <.001	29.6%	1284	150.4 (4), <.001
≥ 2 to < 3	13.0%	3787		19.8%	2723	
≥ 3 to < 4	10.1%	3387		17.5%	3381	
≥ 4 to < 6	9.6%	2865		15.1%	4499	
≥ 6	13.1%	747		14.4%	1858	
Average daily intakes of pure fruit juice						
No consumption	12.8%	3430	0.63 (2), .730	17.5%	5334	12.6 (2), .002
≤ 1 per day	12.4%	9613		17.4%	8214	
> 1 per day	13.4%	374		27.4%	197	
Average daily intakes of salty snacks						
No consumption	12.7%	2385	4.13 (2), .127	16.9%	2742	0.97 (2), .615
> 0 to ≤1	12.4%	11,004		17.7%	10,973	
> 1 to ≤10	25.0%	28		20.7%	30	
Average daily intakes of calcium sources with high vitamin D content						
> 0 to < 1	13.6%	3007	19.4 (3), <.001	18.7%	3027	6.12 (3), .106
≥ 1 to < 2	11.4%	6227		17.4%	6084	
≥ 2 to < 4	13.1%	3717		16.7%	4130	
≥ 4	16.8%	466		19.5%	504	
Average daily intakes of calcium sources with low vitamin D content						
No consumption	15.2%	3343	26.0 (1), <.001	21.5%	1625	17.8 (1), <.001
> 0	11.7%	10,074		17.1%	12,120	
Average daily intakes of pastries						
No consumption	14.1%	1190	3.16 (2), .206	18.0%	1565	7.75 (2), .021
> 0 to ≤1	12.4%	11,983		17.4%	11,958	
> 1 to ≤13	11.1%	244		24.6%	222	
Average weekly intakes of chocolate bars						
No consumption	11.9%	4542	30.4 (2), <.001	15.8%	4858	38.4 (2), <.001
> 0 to ≤0.6	12.4%	8361		18.0%	8177	
> 0.6	20.4%	514		25.1%	710	

In terms of health-related characteristics, the majority had at least one health condition (*n* = 22,297, 82.1%), some type of hypertension or taking anti-hypertensive medication (*n* = 17,285, 63.6%), and reported having no chronic pain (*n* = 20,569, 75.7%). Most respondents did not drink excess alcohol (*n* = 17,552, 64.6%) and did limited physical activity (*n* = 23,264, 85.6%). There were significant associations between all physical health and health behavior characteristics (p’s < 0.05–0.001). For nutrition status indicators, there tended to be a higher proportion of the sample having characteristics that were indicative of over-nutrition. All body composition measures indicated that the majority tended to have excess weight based on BMI measures (*n* = 18,893, 69.7%), high visceral adiposity based on WHR (*n* = 17,898, 65.9%), high body fat percent (*n* = 16,752, 61.7%), and elevated disease risk based on BMI and WHR measures (*n* = 19,198, 70.8%). There were significant associations for all over-nutrition indications (p’s < 0.05–0.001). While there were significant associations found for indicators of poor nutrition that included grip strength (*p* < 0.001) and nutritional risk (*p* < 0.001), most of the sample did not have the characteristics that would be indicative of under-nutrition. For men,

however, there was a high proportion who had sarcopenia (*n* = 11,282, 41.5% of the total sample) (χ^2^(1) = 57.4, *p* < 0.001). For men, significant association were also found for the anemia screen (χ^2^(2) = 15.6, *p* < 0.001) measurement, however, most of the men screened negative (*n* = 10,448, 38.5% of the total sample).

For both men and women, most of the 12 dietary intake measures were significantly associated with depression. Overall, the sample tended to have low intakes of fiber (≤2 servings/day; *n* = 21,617, 79.6%), however there was only a significant association found for women (χ^2^ = 21.8 (3), *p* < 0.001). Most of the sample also had low intakes of pulses and nuts (≤1 serving/day; *n* = 15,027, 55.3%) as well as fruits and vegetables (≤4 servings/day; *n* = 17,193, 63.3%) and there were significant associations found for both of these dietary intake measures (p’s < 0.001). For the intakes of calcium containing foods, most of the sample consumed low amounts of those with high vitamin D content (≤2 servings/day; *n* = 18,345, 67.5%), and a significant association was only found for men (χ^2^(3) = 19.4, *p* < 0.001). For sources with low vitamin D content, most of the sample consumed some level of these (*n* = 22,194, 81.7%) with significant associations indicated (p’s < 0.001). For dietary sources of the omega-3 fatty acids, most consumed fish (*n* = 24,891, 91.6%, p’s < 0.001); few consumed omega-3 eggs (*n* = 20,058, 73.8%) which only showed significant association for women (χ^2^(1) = 9.6, *p* < 0.05). About half of the sample on average consumed four or more fat sources (*n* = 13,543, 49.9%). There was a significant association between number of fat sources consumed daily and depression among men (χ^2^(3) = 18.1, *p* < 0.001), but not among women. More than two-thirds of the sample’s average daily intakes of pure fruit juice, salty snacks, and pastries were less than once daily and of these pure fruit juice (χ^2^(3) = 12.6, *p* < 0.05) and pastries (χ^2^ (2) = 7.75, *p* < 0.05) showed significant association for women. On a weekly basis, most of the sample on average consumed less than two-thirds of a chocolate bar (*n* = 25,938, 95.5%) and significant association was indicated (p’s < 0.001). The immigrant sample was categorized according to years since immigration with those who had lived in Canada less than 20 years considered as recent and mid-term immigrants. Those who had resided in Canada 20 or more years were considered as long-term immigrants. On average, the recent and mid-term immigrant group had resided in Canada almost 12 years (Mea*n* = 11.8, SD ± 4.6). The average years since immigration for females (Mean = 12.3, SD ± 4.5) was slightly higher than males (Mean = 11.5, SD ± 4.7). Mean years since immigration for the long-term group was 47 years (Mean = 47.1, SD ± 12.1) with the average being slightly higher for males (Mean = 47.3, SD ± 12.1) compared to females (Mean = 46.9, SD ± 12.2).

### Bivariate and multivariable analysis

#### Objective 1. Is immigrant status associated with depression among Canadian women and/or Canadian men aged 45 to 85?

There was a significantly higher proportion of immigrant women with depression compared to Canadian-born women (18.8%, 28.9% versus 17.1%, χ^2^ (2) =31.8, *p* < 0.001) (Please see Table 1). Immigration status and depression were not associated among men (χ^2^(2) = 2.74, *p* = 0.26).

#### Objective 2. To what extent does adjustment for a wide range of demographic, social, economic, and health-related characteristics attenuate the association between immigrant status and depression?

**Fig. 1:**
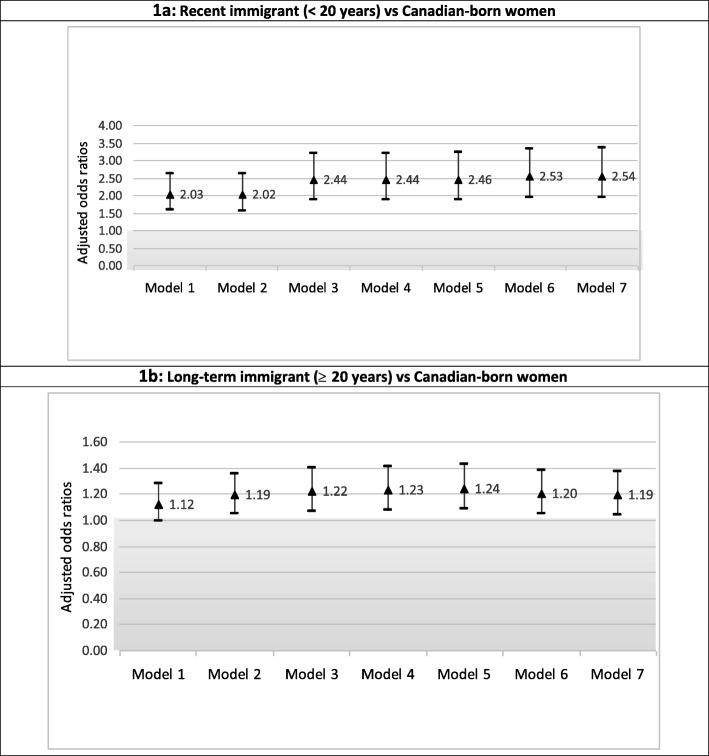
**a** & **b** Adjusted odds ratios of depression by immigration status in females (*N* = 13,745)

**Fig. 2:**
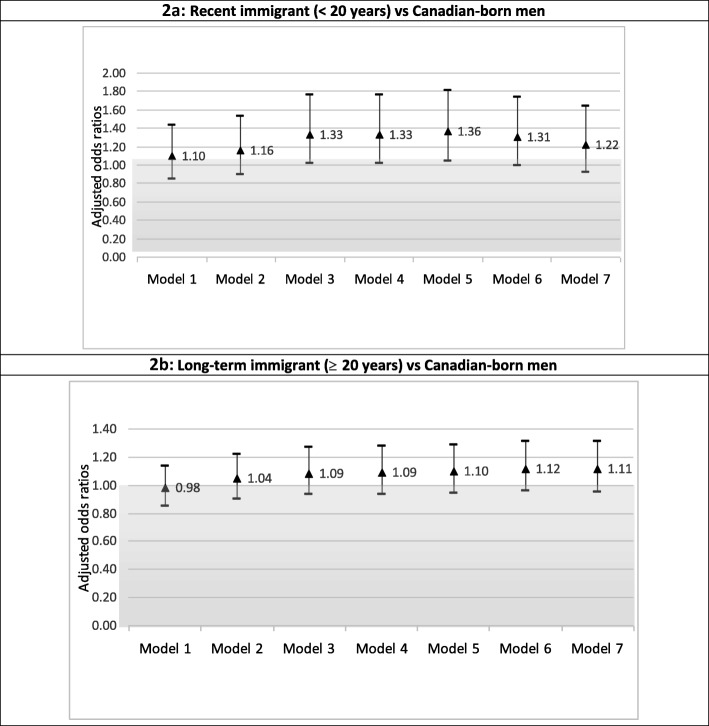
**a** & **b** Adjusted odds ratios of depression by immigration status in males (*N* = 13,417)

As shown in Fig. 1a and b, among women, after adjusting for demographic, social, and economic characteristics, the odds of depression among recent and mid-term immigrant women ranged from 2.03 to 2.54 in comparison to Canadian-born respondents. The odds ratios remained consistently above 2.00 across all models that adjusted for most of the known and/or postulated determinants of depression. The odds of depression among long-term immigrant women ranged from 1.12 (95% CI 0.99–1.27) controlling for demographic, social, and economic factors (Model 1), to 1.19 (95% CI 1.04–1.35) after full adjustments. As shown in Fig. 2a and b, among men, neither recent, mid-term or longer-term immigration status was associated with depression in any of the models.

In sum, the adjusted odds ratios across all models (Figs. 1 and 2) showed that for recent and mid-term immigrant women the likelihood of depression was at least two times that of Canadian-born women. This association between immigrant status and depression among recent and mid-term immigrant women was consistently robust and not attenuated by a wide range of social, economic, and health-related determinants. Furthermore, although the OR for depression and long-term immigrant women was not significant in Model 1, after social, economic, and health-related factors were adjusted, immigrant women had a higher likelihood of depression compared to native-born Canadian women.

#### Objective 3. Is there an association between dietary intakes and depression after controlling for immigration status?

There were many important differences between men and women when the associations between dietary intakes and depression were considered after controlling for immigration status (Please see Table 2). Men who on average consumed higher levels of fat sources (≥4 sources/day; ORs = 0.67–69, p’s < 0.05), lower levels of omega 3 eggs (OR = 0.86, 95% CI 0.76–0.98, *p* < 0.05), and between 3 and 6 daily servings of fruits and vegetables/day (ORs = 0.71–0.74, p’s < 0.05) had a lower likelihood of depression. Men who consumed any chocolate bars on a weekly basis had an increased likelihood of depression (ORs = 1.14–1.72, p’s < 0.05). For women, lower levels of fruit and vegetable intakes (< 2 servings/day, OR = 1.33, 95% CI 1.09–1.64, *p* < 0.05), higher intakes of pure fruit juice (> 1 serving/day, OR = 1.80, 95% CI 1.24–2.59, *p* < 0.05), average daily intakes of 1 or less of salty snacks (OR = 1.19, 95% CI 1.04–1.36, *p* < 0.05), and any chocolate bar consumption (ORs = 1.15–1.66, p’s < 0.05) were significantly associated with depression.

**Table 2:** Adjusted odds ratios of depression by demographic, social, economic, health, nutrition, dietary intake variables (Model 7)

Variable (reference category)	Men		Women	
	aOR (95% CI)	*p-value*	aOR (95% CI)	*p-value*
Demographic, Social, and Economic Characteristics				
Immigration status (Native-born Canadian)				
Immigrant < 20 years	1.22 (0.91, 1.64)	0.18	2.54 (1.93, 3.35)	< 0.001
Immigrant ≥20 years	1.11 (0.95, 1.31)	0.20	1.19 (1.04, 1.37)	0.01
Age (45–55 years)				
56–65 years	0.73 (0.63, 0.84)	< 0.001	0.79 (0.70, 0.90)	< 0.001
66–75 years	0.47 (0.39, 0.57)	< 0.001	0.57 (0.49, 0.67)	< 0.001
76–85 years	0.40 (0.31, 0.50)	< 0.001	0.56 (0.46, 0.69)	< 0.001
Income (≥ $150,000 CAD)				
< $20,000	3.05 (2.26, 4.11)	< 0.001	3.19 (2.47, 4.13)	< 0.001
$20,000-49,999	2.21 (1.79, 2.73)	< 0.001	2.31 (1.91, 2.81)	< 0.001
$50,000-99,999	1.72 (1.44, 2.07)	< 0.001	1.62 (1.37, 1.92)	< 0.001
$100,000-149,999	1.44 (1.19, 1.73)	< 0.001	1.23 (1.02, 1.48)	0.03
Not answered	2.32 (1.73, 3.13)	< 0.001	2.39 (1.90, 3.01)	< 0.001
Marital status (Single)				
Married/living with a partner/ common-law	0.62 (0.52, 0.75)	< 0.001	1.00 (0.85, 1.19	0.96
Widowed/divorced/separated	0.94 (0.75, 1.16)	0.54	0.90 (0.76, 1.08)	0.27
Education level (High school graduate and/or with some post-secondary)				
Less than secondary school	0.92 (0.70, 1.21)	0.54	1.23 (1.00, 1.52)	0.05
Post-secondary degree/diploma	0.82 (0.70, 0.95)	0.01	0.99 (0.87, 1.12)	0.88
Not answered	1.09 (0.30, 3.93)	0.90	0.41 (0.11, 1.55)	0.19
Physical Health				
Morbidities (No conditions)				
1 health condition	1.36 (1.13, 1.65)	< 0.001	1.27 (1.05, 1.53)	0.01
2 health conditions	1.83 (1.51, 2.23)	< 0.001	2.18 (1.83, 2.61)	< 0.001
3 health conditions	3.65 (3.03, 4.40)	< 0.001	3.01 (2.54, 3.57)	< 0.001
Hypertension levels (Normal)				
Elevated	1.02 (0.81, 1.28)	0.88	1.07 (0.90, 1.27)	0.45
Stage 1 hypertension	1.10 (0.94, 1.28)	0.23	1.31 (1.13, 1.51)	< 0.001
Stage 2 hypertension	1.08 (0.89, 1.31)	0.46	1.09 (0.91, 1.30)	0.37
Taking anti-hypertensive	0.92 (0.78, 1.08)	0.30	1.12 (0.98, 1.29)	0.10
Chronic pain (No pain)				
Pain	1.86 (1.63, 2.13)	< 0.001	1.79 (1.60, 1.99)	< 0.001
Refused	2.17 (0.88, 5.38)	0.09	1.35 (0.64, 2.88)	0.43
Health Behaviors				
Smoking ≥100 cigarettes (< 100)	1.17 (1.04, 1.32)	0.01	0.97 (0.88, 1.07)	0.56
Binge drinking (No binge drinking)				
Regular binge drinking	1.03 (0.98, 1.20)	0.66	1.05 (0.91, 1.22)	0.51
Occasional binge drinking	0.91 (0.79, 1.06)	0.22	0.98 (0.86, 1.11)	0.70
Physical activity (Sometimes or often)				
Never or seldom	1.06 (0.87, 1.29)	0.59	1.14 (0.97, 1.35)	0.12
No answer or refused	1.02 (0.36, 2.89)	0.98	0.97 (0.39, 2.44)	0.96
Nutrition Status Indicators				
BMI (Underweight: < 18.5)				
Normal weight: 18.5–24.99	0.58 (0.27, 1.24)	0.16	0.88 (0.55, 1.40)	0.59
Overweight: 25–29.99	1.44 (0.28, 7.38)	0.66	0.66 (0.36, 1.19)	0.17
Obese: 30 or above	0.89 (0.17, 4.76)	0.89	0.48 (0.22, 1.02)	0.06
Waist-to-hip ratio (Low risk)	0.97 (0.79, 1.19)	0.78	0.94 (0.84, 1.06)	0.33
Waist-to-height ratio (Below cut-off)	0.95 (0.81, 1.11)	0.51	1.14 (0.98, 1.32)	0.09
Disease risk (Least risk)				
Increased	0.39 (0.09, 1.64)	0.20	1.32 (0.91, 1.91)	0.14
High	0.41 (0.10, 1.79)	0.24	1.22 (0.80, 1.87)	0.35
Very high	0.61 (0.13, 2.80)	0.53	1.55 (0.82, 2.94)	0.18
Body fat percent (0–26%)				
26–31%	1.03 (0.89, 1.20)	0.70	0.87 (0.61, 1.24)	0.45
31–36%	1.04 (0.85, 1.27)	0.70	0.79 (0.57, 1.10)	0.16
36–41%	1.27 (0.89, 1.82)	0.19	0.86 (0.61, 1.20)	0.37
41–59%	1.15 (0.73, 1.82)	0.54	0.87 (0.61, 1.23)	0.42
Grip strength (No under-nutrition)				
Under-nutrition	1.25 (1.08, 1.43)	< 0.001	1.25 (1.07, 1.45)	< 0.001
Not assessed	1.27 (1.00, 1.63)	0.05	1.08 (0.92, 1.28)	0.34
Nutritional risk (Low risk)				
High risk	2.24 (1.98, 2.53)	< 0.001	2.24 (2.02, 2.49)	< 0.001
Not assessed	1.18 (0.65, 2.14)	0.59	1.95 (1.17, 3.24)	0.01
Skeletal Muscle Index (No sarcopenia)				
Sarcopenia	1.05 (0.80, 1.36)	0.74	0.43 (0.03, 6.33)	0.54
T-scores (Normal bone density)				
Osteoporosis	0.63 (0.19, 2.06)	0.44	1.11 (0.91, 1.37)	0.30
Osteopenia	0.98 (0.76, 1.26)	0.88	1.11 (0.98, 1.24)	0.09
Screen for anemia (Negative)				
Positive	1.16 (0.92, 1.44)	0.20	1.01 (0.56, 1.82)	0.97
No consent for blood work	1.05 (0.91, 1.23)	0.50	0.98 (0.86, 1.11)	0.72
Dietary Intakes				
Average daily intakes of fiber sources (0 to < 1)				
≥ 1 to < 2	1.14 (1.00, 1.30)	0.05	0.93 (0.83, 1.03)	0.17
≥ 2 to < 3	1.04 (0.86, 1.24)	0.71	1.13 (0.97, 1.32)	0.11
≥ 3	1.47 (1.10, 1.95)	0.01	1.17 (0.90, 1.52)	0.25
Average daily intakes of pulses and nuts (0 to < 0.5)				
≥ 0.5 to < 1	0.95 (0.82, 1.10)	0.48	0.98 (0.86, 1.12)	0.74
≥ 1 to < 2	0.94 (0.82, 1.08)	0.41	1.04 (0.92, 1.16)	0.56
≥ 2	1.06 (0.83, 1.36)	0.65	0.91 (0.74, 1.13)	0.40
Average daily intakes of fat sources (0 to < 2.5)				
≥ 2.5 to < 4	0.83 (0.69, 1.00)	0.05	1.00 (0.84, 1.17)	0.96
≥ 4 to < 5	0.67 (0.54, 0.84)	< 0.001	0.91 (0.75, 1.10)	0.34
≥ 5	0.69 (0.54, 0.88)	< 0.001	0.99 (0.81, 1.22)	0.95
Intakes of fish (Consumes)	1.32 (1.11, 1.57)	< 0.001	1.00 (0.85, 1.18)	0.98
Intakes of omega-3 Egg (Consumes)	0.86 (0.76, 0.98)	0.02	1.02 (0.91, 1.14)	0.73
Average daily intakes of fruits and vegetables (≥ 6)				
0 to < 2	0.92 (0.71, 1.19)	0.51	1.33 (1.09, 1.64)	0.01
≥ 2 to < 3	0.87 (0.69, 1.11)	0.28	1.04 (0.88, 1.24)	0.64
≥ 3 to < 4	0.74 (0.58, 0.94)	0.02	1.06 (0.90, 1.25)	0.46
≥ 4 to < 6	0.71 (0.55, 0.91)	0.01	0.97 (0.83, 1.13)	0.71
Average daily intakes of pure fruit juice (No consumption)				
≤ 1 per day	1.01 (0.88, 1.15)	0.94	0.98 (0.89, 1.08)	0.67
> 1 per day	1.05 (0.74, 1.49)	0.78	1.80 (1.24, 2.59)	< 0.001
Average daily intakes of salty snacks (0)				
0 to ≤1	1.04 (0.88, 1.23)	0.63	1.19 (1.04, 1.36)	0.01
> 1 day	2.31 (0.89, 5.96)	0.08	1.09 (0.41, 2.86)	0.86
Average daily intakes of calcium sources with high vitamin D content (≥ 4)				
0 to < 1	0.72 (0.52, 1.02)	0.06	0.95 (0.70, 1.28)	0.74
≥ 1 to < 2	0.73 (0.53, 1.00)	0.05	0.96 (0.72, 1.26)	0.75
≥ 2 to < 4	0.95 (0.71, 1.28)	0.75	0.90 (0.69, 1.17)	0.42
Average daily intakes of calcium sources with low vitamin D content (> 0)				
No consumption	1.02 (0.89, 1.17)	0.75	1.10 (0.95, 1.28)	0.19
Average daily intakes of pastries (0)				
> 0 to ≤1	1.06 (0.87, 1.30)	0.55	0.99 (0.85, 1.16)	0.94
> 1	0.90 (0.57, 1.44)	0.67	1.35 (0.93, 1.95)	0.12
Average weekly intakes of chocolate bars (0)				
> 0 to ≤0.6	1.14 (1.01, 1.30)	0.04	1.15 (1.04, 1.28)	0.01
> 0.6	1.72 (1.32, 2.25)	< 0.001	1.66 (1.35, 2.05)	< 0.001

#### Objective 4. What other factors are associated with depression among Canadians aged 45 to 85 after controlling for immigration status?

The odds of depression for demographic, social, and economic variables differed by gender (Please see Table 2). For men only, those who were married, living with a partner or in a common-law relationship (OR = 0.62, 95% CI 0.52–0.75, *p* < 0.001) or who had a post-secondary degree or diploma (OR = 0.82, 95% CI 0.70–0.95, *p* < 0.05) had a lower likelihood of depression. For both men and women who were 55 years or older, there was a lower likelihood of depression (ORs 0.40–0.79, p’s < 0.001), and an inverse relationship between depression as incomes increased (p’s < 0.001).

Associations between depression and different health determinants such as physical health and health behaviors also varied between men and women. Men who reported having at least one health condition (ORs = 1.36–3.65, p’s < 0.001), having chronic pain (OR = 1.86, 95% CI 1.63–2.13, *p* < 0.001), or smoking (OR = 1.17, 95% CI 1.04–1.32, *p* < 0.001) had a higher likelihood of reporting depression. Women who had least one health condition (ORs = 1.27–3.01, *p*’s < .05), chronic pain (OR = 1.79 95% CI 1.60–1.99, *p* < 0.001), or stage 1 hypertension (OR = 1.31, 95% CI 1.13–1.51, *p* < 0.001) had a higher likelihood of reporting depression.

For nutrition status indicators, men and women tended to have the same factors associated with depression. These included grip strength as a measure of under-nutrition (ORs = 1.25, *p*’s < 0.001) and high nutritional risk (ORs = 2.24, *p*’s < 0.001). For women not assessed for nutritional risk (OR = 1.97, *p* < 0.05), the likelihood of depression was higher.

Depression in the above analyses was based solely upon the individual’s current CES-D scores. Therefore, those with a score below the cutpoint were classified as ‘not depressed’ even if they were taking prescribed antidepressants and/or had been diagnosed with depression. We conducted a sensitivity analysis in which we reclassified these individuals into the ‘depressed’ category. When we re-analyzed the data with this new outcome variable, the findings were similar to the original with respect to relationship between the immigrant variable and depression.

## Discussion

This study examined depression in a nationally representative sample of Canadians in middle and late adulthood and found significant effect modification between immigrant status and sex. For women, depression was associated with immigrant status, less than secondary school graduation education, stage 1 hypertension, chronic pain, low intakes of fruits and vegetables, consumption of pure fruit juice, and intakes of salty snacks. For men, being in a relationship and having a post-secondary degree or diploma appeared to be protective against depression. Conversely, men were more likely to experience depression if they smoked, consumed higher levels of fat, or lower levels of omega 3 eggs, fruits and vegetables, or calcium sources with high vitamin D content. For both men and women, depression was associated with having at least one health condition, pain, poor nutritional status, and consuming chocolate bars.

### Objective 1. Is immigrant status associated with depression among Canadian women and/or Canadian men aged 45 to 85?

Among women, immigrant status was associated with depression. This finding is consistent with longitudinal investigations of new immigrants which show increases in reported sadness and loneliness [29], but is inconsistent with cross-sectional studies that report lower levels of depressive symptoms when compared to native born Canadians [25–28]. Differences in the results may be attributed to the older age of this sample (45–85 years old) and how depression was measured and what potential confounders were accounted for. The immigrant women in this study may have reported depression as a result of the substantial stress associated with settling in a new country such as having insufficient income [40], overcoming language barriers [41, 42], facing discrimination, adapting to a different culture [43], reduced social support networks [44], and having their education and work experiences unrecognized [45].

### Objective 2. To what extent does adjustment for a wide range of demographic, social, economic, and health-related characteristics attenuate the association between immigrant status and depression?

Contrary to the assumption of a health advantage of immigrants, our results showed no association with depression for men and found that middle-aged and older immigrant women are *more* likely to screen positive for depression compared to those who were born in Canada. The association of heightened depression was not attenuated when 32 covariates including socioeconomic status, health behaviors, nutrition status, and dietary habits were taken into account, suggesting that these may not be the main determinants of depression among immigrants. One possible explanation is that based on cumulative advantage-disadvantage theory [88–90], exposure to migration and acculturation may contribute to stress over the life course and poorer mental health status despite having similar socioeconomic status and lifestyles as middle-aged and older non-immigrants.

Our results showed that middle-aged and older immigrant women who lived in Canada less than 20 years have a higher likelihood of depression compared to both long-term immigrant women and native-born Canadian women. Similar findings were also indicated in a longitudinal study where deterioration of self-rated health occurred among females and ethnic minority immigrants over 4 years after arriving in Canada [68]. Our study also suggested that the trajectory of deterioration in mental health among middle-aged and older immigrants might not be a linear relationship. As suggested by Wu and colleagues [28], a reverse U shape association may occur between length of stay and depression among immigrants. This would be consistent with the disillusionment model which suggests that newly arrived immigrants may experience “euphoria of arrival” and have similar or better health status than native-born residents. Subsequently, they enter a period of disillusionment and experience difficulties in the receiving country, and then eventually, adapt to the new environment [91, 92]. Further study examining longitudinal associations will better ascertain these relationships.

### Objective 3. Is there an association between dietary intakes and depression after controlling for immigration status?

Similar to previous research [14–18], the consumption of fruits and vegetables was protective for depression. Various anti-inflammatory and anti-oxidant components in fruits and vegetables may account for this relationship. Several minerals and vitamins (e.g., magnesium, zinc, selenium) present in fruits and vegetables may reduce plasma concentrations of C-reactive protein, a marker of low-grade inflammation associated with depression [93]. Antioxidants, such as vitamin C, vitamin E, and folic acid, are involved in endothelial cell signaling cascades which reduce the effects of oxidative stress on mental health [94, 95].

Interestingly, in this study chocolate intake was associated with depression. Cocoa, which is found in chocolate, has polyphenolic compounds that have been reported to modulate mental health [96] due to their roles in metal ion (e.g., iron, copper) chelation, modulation of antioxidant enzyme antioxidant ability, and anti-inflammation [97]. The reported effects of cocoa with mental health conditions such as depression are still equivocal [98]. It is also important to note that the chocolate assessed in this study referred to all types including milk and dark chocolate; the latter has 2–3 times more of the beneficial flavanol-rich cocoa. Furthermore, the relationships between chocolate consumption and depression may be impacted by whether it is consumed mindfully or not [99]. Finally, the cross-sectional nature of this study prohibits the determination of causality. Some individuals crave chocolate and its self-soothing qualities when they are depressed [100].

For men, high fiber intakes were associated with depression. High fiber foods contain phytates which bind with trace minerals such as iron, zinc, and manganese and reduces their bioavailability [101]. With increased high fiber intakes less of these nutrients, which are critical for mental health, may be absorbed which can subsequently contribute to depression symptoms [102]. Dietary fiber intake can alter the human gastrointestinal (GI) microbiota, and GI microbe composition is associated with mood in adults. Recent research has suggested that these relationships differ by sex [103].

Surprisingly, higher fat intakes tended to be protective for depression in men. This finding, which seems counterintuitive, may be due to the fact that the measure of fat included all fatty acid types and did not partition them into saturated and unsaturated fat; each type has different relationships with depression [104]. Recent exploratory research suggests that the absorption of bile acid may be impaired in chronic stress [105–107] and that intakes of high-fat diets under stress may induce cholesterol metabolism and help to attenuate stress-related outcomes [108].

Similar to previous findings [104], intakes of omega-3 polyunsaturated fats were inversely associated with depression, however this relationship was only shown for men. This appears to be the first study to suggest the relationship between omega-3 fatty acid intake and depression may differ by gender. However, previous investigations have found sex differences for depression outcomes based on the ratio of polyunsaturated fat to saturated fat consumed [109]. Increased omega-3 fatty acid concentration in the diet may influence central nervous system cell membrane fluidity, and phospholipid composition, which may alter the structure and function of the embedded proteins and affect serotonin and dopamine neurotransmission [110].

The disproportionate associations of fruit juice and salty snack intakes with depression found in women may be due to their heightened susceptibility to factors that promote inflammation [111]. The high content of naturally occurring sugars in 100% fruit juice may cause negative health effects similar to those of other sugar-sweetened beverages [112]. Some studies have indicated that sodium reabsorption may be associated with psychological activity. Specifically, the inhibition of renal sodium reabsorption is compensated for by stimulation of the salt appetite and vice versa [111]. Exposure to stress can activate the hypothalamic-pituitary-adrenal (HPA) axis leading to release of corticosteroids which bind to two types of receptors in the brain: the mineralocorticoid receptor and the glucocorticoid receptor. Individuals with low mineralocorticoid receptor function may be more susceptible to depression [113].

### Objective 4. What other factors are associated with depression among Canadians aged 45 to 85 after controlling for immigration status?

Consistent with previous research, our results support that among middle-aged and older adults, those who are women, working-age (45–65 years), married, have lower education, and have less annual income are more likely to experience depression. Previous research has consistently shown that being female and being unmarried are associated with more depressive symptoms [114, 115], while research findings about the interaction between sex and age on depressive symptoms remained inconsistent [116, 117]. The association of depression with lower socioeconomic status is consistent across both cross-sectional investigations [3, 118] and longitudinal research of older adults in the Netherlands [119] and examination of depression and education attainment over the life course in the US [120]. Our study provides further evidence of the gradient effect between income level and depression.

In addition to demographic, social, and economic characteristics, associations between physical health factors and depression were also supported by previous findings. Consistent with a meta-analysis focusing on older adults, presence of chronic disease is an independent risk factor of depression [121]. Longitudinal studies of older adults also indicate that experiencing pain is associated with depression [122]. Having other physical conditions, such as hypertension, are also associated with depression among older adults [123].

With the exception of the association between smoking and depression in men, our findings of no other significant associations between depression and health behaviors were not consistent with previous studies [124–126]. Other investigations have indicated that the associations found between smoking and depression and between smoking and heavy drinking are bidirectional [127]. The different results may be due to the variability in measures of substance use that were administered. The lack of association found between physical activity and depression may be due to confounding by factors such as presence of a health condition or having an insufficient sample who were really active [128].

The relationships between depression and nutrition status indicators were both similar and dissimilar to other studies. Like other investigations, our bivariate analyses showed association between excess body weight and depression. However, the multivariable logistic regression results did not show this relationship. This may suggest that the association was attenuated by other factors such as presence of health conditions, or, as indicated by other studies, the relationship between BMI and depression in older adults may be curvilinear [129]. Similar to previous studies, measures of poor nutrition status were associated with depression in older adults [130]. Poor nutrition is closely related to frailty, which can impact overall physical and psychological functioning [131].

Generally speaking, for women, more factors tended to be associated with depression. This may be due to women having heightened susceptibility to inflammation and autoimmune responses [132, 133] which can elevate risk for depression [134, 135]. For women, inflammation tends to affect their feelings of social disconnection to a greater extent than men [136]. In addition, factors such as relationship distress and obesity, which are both associated with depression risk, tend to have greater association with inflammation for women than for men [137]. Women experience several risk factors for inflammation at higher rates than men, including low physical inactivity and childhood adversity [105, 111, 138]. The fluctuation of women’s reproductive hormones throughout the life span, also has implications for inflammation and depression. For example, during the menopausal transition, women experience pronounced hormonal fluctuations which result in diminished estrogen levels following menopause [132, 133] and elevated levels of inflammation [134, 135]. Collectively, these findings suggest that women’s susceptibility to inflammation can contribute to the observed sex differences for depression.

To the best of our knowledge, this is the first Canadian study to comprehensively assess associations between depression and various health determinants, including immigration status and nutritional intake. However, the interpretation of the results should be viewed with caution. Given that the data was cross-sectional, the direction of the relationships between depression and the various measures cannot be ascertained. There are many patho-physiological processes that are involved in depression and thus it was impossible to account for all variables that may affect the relationships between depression and different health determinants. The use of self-report measures for many of the variables made misreporting and misclassification possible. This may be particularly the case for the lifetime measure used for smoking status and for the physical activity measurement which only assessed activity in the previous 7 days. Unfortunately, due to limited sample size of relatively recent and mid-term immigrants, larger cohorts of immigrants were analyzed (i.e., < 20 years and ≥20 years) and therefore relationships within more defined sub-groups could not be examined. Suggestions for future investigations include oversampling different immigrant groups to enable sub-population analysis, examining longitudinal data to explore relationship trajectories between depression and health determinants post-settlement, and conducting qualitative work to gain important insights into the quantitative findings.

## Conclusions

Depression is a leading cause of disability worldwide. In this study, older immigrant women were more susceptible to depression when compared to Canadian-born women and all men. The differences between women and men may be due to dissimilarities in their susceptibility to inflammatory and autoimmune processes. Successful prevention and treatment of depression among older adults, particularly immigrant women, could have major public health, societal, and economic impacts. Based on our findings, interventions that target social, economic, physical health, and nutrition-related factors that can mitigate inflammatory responses may be particularly important in improving the mental health of older adults. This investigation provides important insights for policy and program development to mitigate depression in older adults, particularly for marginalized groups such as immigrant women.

## Data Availability

Data are available from the Canadian Longitudinal Study on Aging (www.clsa-elcv.ca) for researchers who meet the criteria for access to de-identified CLSA data.

## Abbreviations

AB SCREENTM II: Abbreviated Seniors in the Community Risk Evaluation for Eating and Nutrition II
aOR: Adjusted odds ratios
BMDs: Bone mineral densities
BMI: Body mass index
CES-D-10: Center for Epidemiologic Studies Short Depression
CI: Confidence interval
CLSA: Canadian Longitudinal Study on Aging
DEXA: Dual-energy x-ray absorptiometry
g/L: grams/Litre
mm Hg: Millimetres mercury
OR: Odds ratio
SD: Standard deviation
WHR: Waist-to-hip ratio
WHtR: Waist-to-height ratio
